# A Malignant Neoplasm Imitating a Subcutaneous Cyst

**Published:** 2012-07-20

**Authors:** Daisuke Maeda, Kyosuke Minami, Yoko Osaki, Hideaki Miwa, Ko Hosokawa, Tateki Kubo

**Affiliations:** Osaka Rosai Hospital, Sakai, Japan

## DESCRIPTION

**Figure 1:**
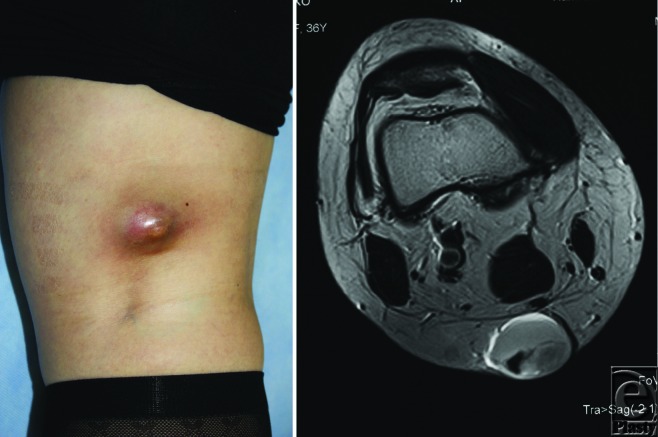
The tumor in the right popliteal fossa (naked eye appearance and MRI).

A 36-year-old woman presented with a cystic lesion located on the right popliteal fossa, which grew slowly for 7 years and became remarkably larger in the last few months. The lesion was dark red in color (Fig 1) and elastic hard in consistency. When punctured by her primary care physician, darkish blood was aspirated.

## QUESTIONS

1. **What is the differential diagnosis of a subcutaneous cyst around the knee?**
2. **Which diagnostic imaging studies are appropriate to help reach diagnosis?**
3. **What are the therapeutic options for this lesion?**

## DISCUSSION

The differential diagnosis of a lesion presenting as a subcutaneous cyst includes the following benign entities: epidermoid cyst, calcifying epithelioma, venous malformation, lymphatic malformation, hemangioma, ganglion, and abscess. Especially, Baker's cyst, known as a popliteal cyst, is frequently seen in the popliteal area, reportedly in 10% to 41% of MR (magnetic resonance) examinations of the knee.^1^ The diagnosis of Baker's cyst is effectively made with MR imaging (MRI) because fluid distention of the gastrocnemius semimembranosus bursa is well depicted.^2^ Epidermoid cyst is one of the most common cystic lesion in the skin. It is usually mobile over deeper structures and hardly involves surrounding skin unless it becomes infected. Moreover, malignant lesions, such as dermatofibrosarcoma protuberans (DFSP), liposarcoma, malignant fibrous histiocytoma, malignant peripheral nerve sheath tumor, and fibrosarcoma, should be considered.^3^^,^^4^ Biopsy examination revealed that our patient had DFSP; it is an intermediate malignant neoplasm, which typically originates from the dermis and presents as a protuberant nodular cutaneous mass. However, deep DFSP may present as a subcutaneous mass with exclusive or near-exclusive subcutaneous involvement, and hemorrhage and cystic change may occur as in this case.^5^ For definite diagnosis, biopsy must be made as with all other solid tumors.

**Figure 2:**
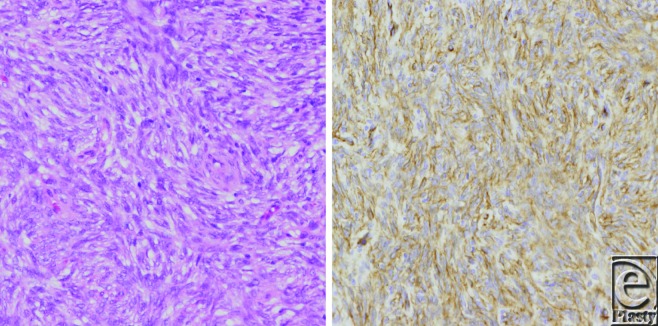
CD34-positive spindle-shaped cells. Hematoxylin and eosin staining (*left*) and CD34 staining (*right*).

In biopsy planning, care must be taken not to jeopardize subsequent treatment. There should be no rising of flaps for wound closure, or contamination of surrounding tissues with tumor cells.^6^ Preferably, the surgeon performing the biopsy will be the same person who will perform the definitive procedure.^7^ Hematoxylin and eosin staining of DFSP shows a storiform proliferation of monomorphic, hyperchromatic spindle-shaped cells. Other histological changes that may be detected include myxoid change, perivascular myxoid nodules, and fibrosarcomatous progression.^8^ Immunohistochemistry reveals CD34 and vimentin-positive cells (Fig 2).

Diagnostic imaging studies of DFSP have not been established, but MRI^9^ and color Doppler ultrasonography^10^ may be useful. Especially, MRI (Fig 1) is helpful to evaluate the depth of infiltration.^9^ For management of DFSP, complete removal of the tumor by Mohs surgery or traditional wide excision should be considered at the time of initial treatment.^11^^,^^12^ Histological assessment of all surgical margins is important before reconstructive procedure. Radiation is sometimes used as a postoperative adjuvant therapy for positive surgical margins.^13^ However, re-resection should be considered whenever possible. Recently, Imatinib mesylate, platelet-derived growth factor receptor inhibitor as a molecularly targeted treatment, has been reportedly useful for adult patients with unresectable, recurrent, or metastases of DFSP with translocation between chromosomes 17 and 22.^14^
